# A bibliometric review of oncolytic virus research as a novel approach for cancer therapy

**DOI:** 10.1186/s12985-021-01571-7

**Published:** 2021-05-12

**Authors:** Amir Sasan Mozaffari Nejad, Tehjeeb Noor, Ziaul Haque Munim, Mohammad Yousef Alikhani, Amir Ghaemi

**Affiliations:** 1 Research Center for Molecular Medicine, Hamadan University of Medical Sciences, Hamadan, Iran; 2 Faculty of Medicine, University of Bergen, Horten, Norway; 3 Faculty of Technology, Natural and Maritime Sciences, University of South-Eastern Norway, Horten, Norway; 4 Department of Microbiology, Faculty of Medicine, Hamadan University of Medical Sciences, Hamadan, Iran; 5 Department of Influenza and Other Respiratory Viruses, Pasteur Institute of Iran, Tehran, Iran

**Keywords:** Cancer, Oncolytic virus, Virotherapy, Bibliometric, Dynamic co-citation

## Abstract

**Background:**

In recent years, oncolytic viruses (OVs) have drawn attention as a novel therapy to various types of cancers, both in clinical and preclinical cancer studies all around the world. Consequently, researchers have been actively working on enhancing cancer therapy since the early twentieth century. This study presents a systematic review of the literature on OVs, discusses underlying research clusters and, presents future directions of OVs research.

**Methods:**

A total of 1626 published articles related to OVs as cancer therapy were obtained from the Web of Science (WoS) database published between January 2000 and March 2020. Various aspects of OVs research, including the countries/territories, institutions, journals, authors, citations, research areas, and content analysis to find trending and emerging topics, were analysed using the bibliometrix package in the R-software.

**Results:**

In terms of the number of publications, the USA based researchers were the most productive (n = 611) followed by Chinese (n = 197), and Canadian (n = 153) researchers. The *Molecular Therapy* journal ranked first both in terms of the number of publications (n = 133) and local citations (n = 1384). The most prominent institution was Mayo Clinic from the USA (n = 117) followed by the University of Ottawa from Canada (n = 72), and the University of Helsinki from Finland (n = 63). The most impactful author was Bell J.C with the highest number of articles (n = 67) and total local citations (n = 885). The most impactful article was published in the *Cell* journal. In addition, the latest OVs research mainly builds on four research clusters.

**Conclusion:**

The domain of OVs research has increased at a rapid rate from 2000 to 2020. Based on the synthesis of reviewed studies, adenovirus, herpes simplex virus, reovirus, and Newcastle disease virus have shown potent anti-cancer activity. Developed countries such as the USA, Canada, the UK, and Finland were the most productive, hence, contributed most to this field. Further collaboration will help improve the clinical research translation of this therapy and bring benefits to cancer patients worldwide.

## Background

Cancer is a dreadful disease and one of the leading causes of morbidity and mortality worldwide. According to the latest assessment on cancer’s global burden of cancer by the International Agency for Research on Cancer (IARC) in September 2018, the number of cancer patients has risen by 18.1 million new cases, and 9.6 million deaths [1]. Some of the factors behind the growing cancer burden can be population growth and ageing, prevalent reasons linked to the socio-economic development, and improvement in medical diagnostic procedures. According to a recent study, among the continents, Asia accounted for almost fifty percent of new cases and more than half of the cancer death [2]. There are several therapeutic procedures for cancer treatment, including chemotherapy, radiotherapy, targeted therapy, surgery, stem cell transplant, hormone therapy, and precision medicine. The therapy protocol depends on the site and staging of cancer, patient profile, and availability, among other factors [3, 4]. Most cancer treatment modules are reported to have adverse effects leading to unsatisfactory quality of life and death. Thus, research for new treatment options, limiting the adverse effects, improving life quality during and after treatment, and increasing the efficacy, is ongoing for several years [5]. Oncolytic virotherapy is one of the recent developments in the treatment of cancer.

Oncolytic viruses (OVs) are a novel treatment modality that uses natural or genetically modified (GM) viruses, which, upon infection, selectively replicate and kill neoplastic cells without any severe effects on normal cells. Generally, OVs fall into two categories. The first category includes viruses that normally replicate rather in cancer tissue and are non-pathogenic in humans, such as, autonomous parvoviruses, Seneca Valley virus (SVV), myxoma virus, Reovirus (respiratory enteric orphan), and Newcastle disease virus (NDV). The other type includes viruses that are genetically engineered and/or genetically manipulated, such as vaccinia virus, poliovirus, adenovirus, measles virus, vesicular stomatitis virus (VSV), herpes simplex virus (HSV), and Zika virus [6–9].

There are several studies on OVs and their applications. In the present study, we use the bibliometric analysis method to examine the growth of studies on OVs. We extract bibliography data from the Web of Science (WoS) database from 2000 to March 2020. This study maps the overall research domain on the application of OVs as cancer therapy and extracts future research directions to guide further development in the field.

## Review of bibliometric studies

Bibliometric analysis refers to the study of bibliographic information on published articles. As bibliometric analysis relies on statistical methods, it has emerged as a useful tool to assess the scientific publications in terms of quality and credibility. One of the most common bibliometric tools is the number of citations, which indicates the number of times an article has been cited by other articles. This method aims to identify the most impactful authors, institutions, countries, and journals within a defined subject area.

Studies using bibliometric analysis tools are common in the field of medical studies. For instance, Zou et al. [10] conducted the first study of OVs using data from January 2000 to December 2018; whereas, this study will broaden the coverage up to two more years (data until March 2020). The previous study listed impactful journal, author, country and institutions. In addition to these, this study contributes by mapping the intellectual structure of the field through dynamic co-citation analysis. Unlike the present study, previous studies focused only on the type of cancer diseases [11], prevention of cancer [12], basic epidemiologic methods [13], and some ecologic studies [14, 15] focusing only on a specific country [14, 15], community, or neighbourhood. This study will help oncologists deepen their understanding of the ongoing application of oncolytic viruses in cancer patients worldwide.

## Methodology

**Fig. 1:**
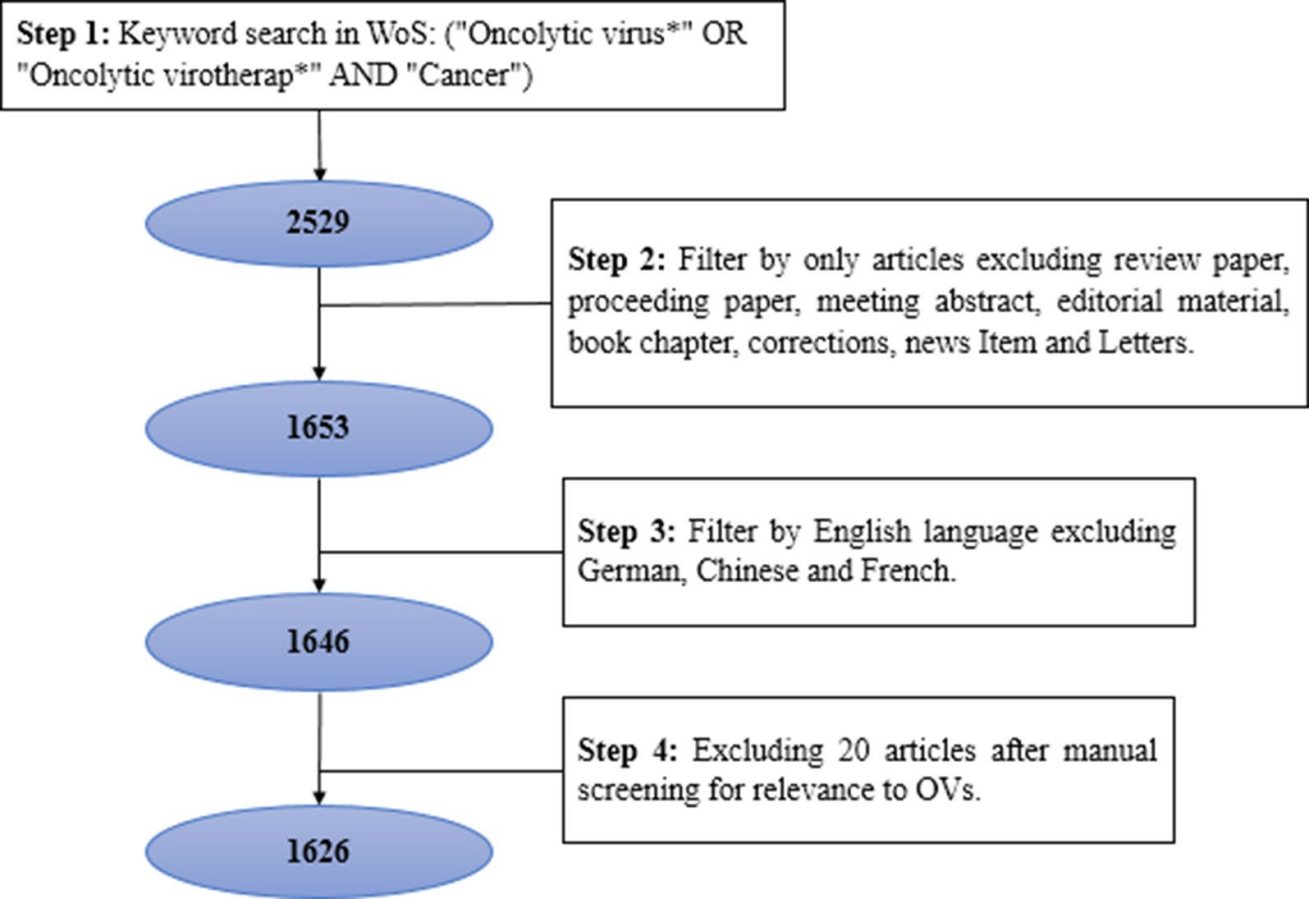
Step-by-step literature search process

On March 2020, the literature search was conducted for relevant articles on the WoS database with the Boolean operator ((“Oncolytic virus*” OR “Oncolytic virotherap*”) AND “Cancer”)). The search resulted in 2529 articles, which were further refined to 1653 articles after excluding review studies (675), proceedings (28), meeting abstracts (80), editorial materials (66), book chapters (17), corrections (4), news items (4) and letters (2). Limiting only to the English language, the articles reduced to 1646 by excluding German (3), Chinse (2), and French (2). We manually reviewed the titles and abstracts of 1646 articles for relevance to our topic of interest and excluded 20 articles. Thus, the final sample for bibliometric analysis included 1626 articles published in 346 academic journals written by 7093 authors during the period of 2000–2020. Figure 1 presents the four steps filtering process. Once the sample was determined, we proceeded with citation and co-citation analysis using the Bibliometrix package [16] in the R-software. Only 32 articles were written by single authors, and on average, each article has 8.17 co-authors. In this study, we analyse previously published data and therefore did not need ethical approval.

## Bibliometric analysis results

### Publication trends

**Fig. 2:**
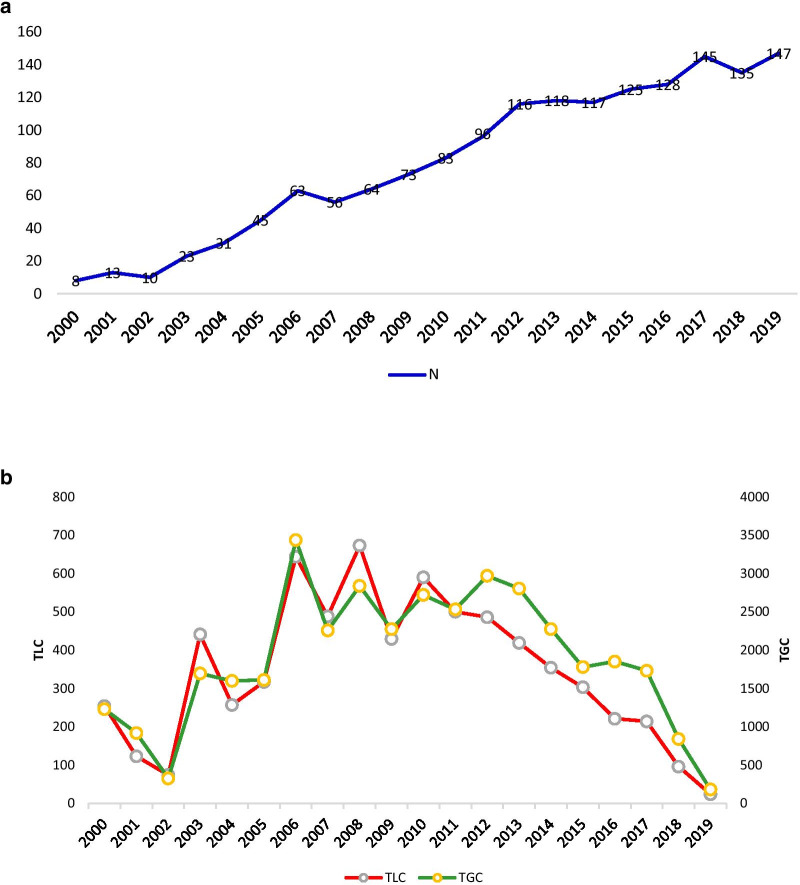
Publication trends (excluding the year 2020). **(a)** Number of studies on OVs during 2000 and 2019, where N represents number of publications, and **(b)** Citations per year during 2000 and 2019, where TLC and TGC represent Total Local Citations and Total Global Citations, respectively

The Bibliometric analysis method is useful in recognizing publication and citation trends in a field of study. As shown in Fig. 2a, we observed the number of publications grew gradually over the last 20 years. A few periods had steady growth such as 2002–2006, 2007–2012 and 2014–2017. Apart from a slight reduction in 2007, 2017 and 2018, in comparison to 2000, the number of publications have increased more than 18 folds in 2019. During this period two drugs, Talimogene laherparepvec (T-Vec) and Oncorine (H101), were approved by the US Food and Drug Administration (FDA) and the China FDA [17] and some OVs entered Phase III clinical trials. Meanwhile, Fig. 2b reports Total Local Citations (TLCs) and Total Global Citations (TGCs). TLCs indicate citations received by the sample of 1626 studies, and TGC indicates citations by all records indexed by WoS. The trends in both TLC and TGC are identical and show an overall lower citation in recent years. This is logical as it takes time for an article to make an impact and get citations after publication.

### Top journals

The top academic journals, based on both the number of publications and the TLCs, are reported in Table 1. The top 20 academic journals which have published more than 15 articles on OVs topic include the journal *Molecular Therapy* (N = 133), *Cancer Gene Therapy* (N = 97) and *Journal of Virology* (N = 75). The top 20 journals with the highest contribution to OVs research published 50.86% of sample of 1626 studies. *Molecular Therapy* achieved the highest number of TLCs (1384), followed by *Cancer Research* (680) and *Nature Medicine* (418). The TGCs are noticeable as it indicates the potential of the publications beyond the OVs’ research domain.

**Table 1:** Top 20 journals in OV research

Rank^a^	Journal	N	TLC	TGC	Rank^b^	Journal	N	TLC	TGC
1	Molecular Therapy	133	1384	5947	1	Molecular Therapy	133	1384	5947
2	Cancer Gene Therapy	97	383	2091	2	Cancer Research	60	680	3287
3	Journal of Virology	75	268	1293	3	Nature Medicine	10	418	1967
4	Cancer Research	60	680	3287	4	Clinical Cancer Research	52	403	1806
5	Gene Therapy	58	375	1788	5	Cancer Gene Therapy	97	383	2091
6	Molecular Therapy-Oncolytics	56	15	292	6	Gene Therapy	58	375	1788
7	Clinical Cancer Research	52	403	1806	7	Journal of Virology	75	268	1293
8	Human Gene Therapy	43	197	935	8	Proceedings of The National Academy of Sciences of The United States of America	14	203	895
9	International Journal of Cancer	42	196	898	9	Human Gene Therapy	43	197	935
10	Oncotarget	39	92	515	10	International Journal of Cancer	42	196	898
11	Plos One	37	0	695	11	Cancer Cell	6	194	966
12	Viruses-Basel	20	4	101	12	Oncotarget	39	92	515
13	BMC Cancer	18	0	345	13	Current Opinion In Molecular Therapeutics	15	83	391
14	Scientific Reports	18	0	139	14	Virology	18	82	481
15	Virology	18	82	481	15	Journal of Theoretical Biology	11	76	144
16	International Journal of Oncology	16	42	224	16	Journal of Clinical Investigation	5	75	225
17	Oncoimmunology	16	0	144	17	Oncogene	13	71	354
18	Current Opinion In Molecular Therapeutics	15	83	391	18	Molecular Cancer Therapeutics	12	65	431
19	Journal of Gene Medicine	15	48	299	19	Cell	1	58	313
20	Journal of Translational Medicine	15	0	211	20	Nature Biotechnology	3	57	319

*TLC* Total local citation, *TGC* total global citation

^a^Ranking based on number of publications

^b^Ranking based on total local citations

### Top institutions

The top 20 contributing institutions in OVs research are presented in Table 2, including the number and percentage of publications from institutions, and their TLC and TGC. Mayo Clinic has published the most studies on OVs with 117 articles and covered 7.2% of the whole literature on this basis, followed by the University of Ottawa (N = 72, 4.4%) and University of Helsinki (N = 63, 3.9%). In relation to the country profile, institutes in the United States of America (USA) has the majority of the top 20 institutions (9/20), and the others are distributed between Canada (5/20), the United Kingdom (UK) (3/20), Germany (2/20), and Finland (1/20).

**Table 2:** Top 20 institutions in OV research

Rank^a^	Institution	N	Percent (%)	TLC	TGC
1	Mayo Clinic	117	7.2	929	3728
2	University of Ottawa	72	4.4	847	3433
3	University of Helsinki	63	3.9	308	1724
4	University of California San Diego	53	3.3	196	1178
5	Ohio State University	52	3.2	499	2650
6	University of Wurzburg	52	3.2	165	873
7	Memorial Sloan Kettering Cancer Center	46	2.8	271	1392
8	University of Pittsburgh	45	2.8	335	1725
9	Institute of Cancer Research	44	2.7	379	1574
10	McMaster University	44	2.7	419	1725
11	Genelux Corporation	42	2.6	140	722
12	German Cancer Research Center	42	2.6	168	940
13	Harvard University	38	2.3	267	1655
14	University of Calgary	34	2.1	278	1141
15	Ottawa Hospital Research Institute	33	2	222	1221
16	University of Surrey	31	1.9	282	1182
17	Massachusetts General Hospital	30	1.8	223	1255
18	Oncolytics Biotech Inc	30	1.8	238	973
19	University of Florida	28	1.7	107	564
20	University of Leeds	28	1.7	341	1200

*TLC* Total local citation, *TGC* total global citation

^a^Ranking based on number of publications per university

### Most impactful authors

Table 3 presents the top 20 authors in the domain of oncolytic virus research, where twenty authors have at least 10 articles in the field of OVs. Bell J.C has the highest number of articles (67) and total local citations (885), followed by Hemminki A. (51 documents and 278 TLC), Russell S.J (48 documents and 385 TLC), and Kanerva A. (39 documents and 25 TLC).

**Table 3:** 20 most impactful authors in OV research

Rank^a^	Author	N	TLC	TLC/t	TGC	TGC/t	TLCb	TLCe
1	Bell JC	67	885	77.93	3598	374.78	161	111
2	Russell SJ	48	385	40.62	1684	176.11	101	57
3	Stojdl DF	19	566	39.87	1970	159.31	64	70
4	Atkins H	15	576	38.78	2041	143.11	61	68
5	Lichty BD	23	416	36.62	1621	160.66	87	70
6	Vile R	27	328	32.94	1225	131.04	109	51
7	Kirn DH	14	347	32.74	1400	137.55	68	58
8	Melcher A	30	328	31.9	1159	119.88	117	34
9	Kottke T	23	313	29.36	1064	105.28	115	32
10	Thompson J	22	313	29.36	1027	102.28	115	32
11	Selby P	21	294	29.03	1112	114.99	88	49
12	Breitbach CJ	10	253	28.2	1008	118.33	65	51
13	Hemminki A	51	278	27.72	1515	156.7	87	34
14	Coffey M	33	257	27.28	1101	126.75	65	42
15	Peng KW	33	252	27.24	1216	130.69	68	36
16	Harrington K	25	259	26.9	1024	111.58	84	33
17	Thorne SH	27	280	25.53	1305	121.5	72	32
18	Diaz RM	19	283	25.49	950	91.05	108	27
19	Kanerva A	39	250	24.67	1313	132.12	76	30
20	Cerullo V	32	189	24.67	942	125.51	62	26

*TLC* Total local citation, *TGC* total global citation, *TLCb* total local citation in the beginning, *TLCe* total local citation in the ending

^a^Ranking based on total local citations per year (TLC/t)

### Most impactful articles

Table 4 presents the top 20 locally cited articles in OVs research during 2000–2020. The ranking is based on the total local citations per year (TLC/t). The table also presents total global citation per year (TGC/t), list of journals publishing the most impactful articles, and their cited reference.

**Table 4:** 20 most impactful articles in OV research

Rank^a^	Authors	Journal	Publication year	Country (authors from)	TLC	TLC/t	TGC	TGC/t	LCR	CR
1	Ribas et al. [18]	Cell	2017	USA, Spain, Switzerland, Australia	58	14.5	313	78.25	1	25
2	Heo et al. [19]	Nature Medicine	2013	USA, Canada, South Korea, Italy	85	10.63	381	47.63	6	22
3	Stojdl et al. [20]	Cancer Cell	2003	USA, Canada	156	8.67	543	30.17	3	51
4	Stojdl et al. [21]	Nature Medicine	2000	Canada	165	7.86	556	26.48	0	21
5	Russel et al. [22]	Mayo Clinic proceedings	2014	USA	39	5.57	149	21.29	4	19
6	Breitbach et al. [23]	Molecular Therapy	2007	USA, Canada	64	4.57	178	12.71	6	46
7	Puzanov et al. [24]	Journal of Clinical Oncology	2016	USA	22	4.4	177	35.4	0	32
8	Chiocca and Rabkin [6]	Cancer Immunology Research	2014	USA	30	4.29	131	18.71	14	55
9	Engeland et al. [25]	Molecular Therapy	2014	USA, Germany	30	4.29	128	18.29	4	27
10	Fulic et al. [26]	Proceedings of the National Academy of Sciences	2006	USA	64	4.27	237	15.8	4	39
11	Freeman et al. [27]	Molecular Therapy	2006	Israel	56	3.73	197	13.13	3	48
12	Parato et al. [28]	Molecular Therapy	2012	USA, Canada, South Korea	33	3.67	126	14	6	46
13	Thorne et al. [29]	Journal of Clinical Investigation	2007	USA, UK, Canada, South Korea	51	3.64	145	10.36	2	23
14	Prestwich et al. [30]	Clinical Cancer Research	2009	USA, UK	43	3.58	105	8.75	10	37
15	Kim et al. [31]	Molecular Therapy	2006	USA, UK, South Korea	50	3.33	180	12	3	48
16	Nguyen et al. [32]	Proceedings of the National Academy of Sciences	2008	USA, Canada	43	3.31	118	9.08	10	50
17	Cerullo et al. [33]	Cancer Research	2010	Finland	36	3.27	131	11.91	4	49
18	Liu et al. [34]	Molecular Therapy	2008	USA, South Korea	42	3.23	143	11	3	19
19	Heise et al. [35]	Nature Medicine	2000	USA, UK	67	3.19	453	21.57	0	33
20	Qiao et al. [36]	Clinical Cancer Research	2008	USA, UK, Canada	41	3.15	119	9.15	5	50

*TLC* Total local citation, *TGC* total global citation, *TGC/t* total global citation per year, *LCR* local cited reference, *CR* cited reference

^a^Ranking based on TLC per year (TLC/t)

As shown in Table 4, the most highly cited article was published in the journal *Cell* in 2017 by Ribas et al. [18] and topped the lists of TLC/t (14.5) and TGC/t (78.25). The second article was published in the journal of *Nature Medicine* by Heo et al. [19], with 10.63 TLC/t and 47.63 TGC/t. Finally, Stojdl et al. [20], ranked third with 8.67 TLC/t and 30.17 TGC/t.

Amongst these top 20 articles, six were published in the journal of *Molecular Therapy*, three were published in *Nature Medicine*, two were published in the *Proceedings of the National Academy of Sciences,* and two were published in *Clinical Cancer Research*. On the other hand, as shown in Table 4 some journals including *Cancer Cell*, *Journal of Clinical Oncology*, *Cancer Immunology Research*, *Journal of Clinical Investigation*, *Cell*, *Cancer Research*, and *Mayo Clinic proceedings,* have published one of the top articles. Also, four articles had authors only from the USA; one article had just one country contribution authors such as Canada, Israel, and Finland. The remaining 13 articles had authors from more than two countries, meaning they resulted from international cooperation.

### Most relevant countries

Table 5 reports the 20 most relevant countries in terms of the number of publications. The USA (611 articles), China (197), and Canada (153) are the three most productive countries in OVs research. The majority of the articles published by the USA based researchers are single country publication (SCP) without having co-authors from other countries. Multiple Country Production (MCP) ratio indicates that authors based in Iran, Belgium, Austria, and France have collaboration worldwide.

**Table 5:** Top 20 most relevant countries

Rank^a^	Country	Articles	SCP	MCP	MCP_Ratio
1	USA	611	442	169	0.28
2	China	197	153	44	0.22
3	Canada	153	104	49	0.32
4	Germany	132	65	67	0.51
5	Japan	109	84	25	0.23
6	United Kingdom	93	39	54	0.58
7	Finland	63	28	35	0.56
8	Spain	41	29	12	0.29
9	Korea	28	17	11	0.39
10	Italy	26	13	13	0.50
11	France	22	9	13	0.59
12	Netherlands	18	9	9	0.50
13	Australia	14	8	6	0.43
14	Iran	12	4	8	0.67
15	Russia	11	8	3	0.27
16	Sweden	11	6	5	0.45
17	Austria	10	4	6	0.60
18	Malaysia	10	5	5	0.50
19	Belgium	6	2	4	0.67
20	India	6	5	1	0.17

*SCP* Single country production, *MCP* multiple country production

^a^Ranking based on number of articles

## Intellectual structure of the research domain

In this study, we use dynamic co-citation method for mapping the intellectual structure of the OVs research field. When two or more articles are cited together by other articles, they are called co-cited [37]. Co-cited articles are likely to share the same concepts as they were cited together by other studies. Hence, co-citation analysis allows us to map the intellectual structure of a research domain. Co-citation can also recognize knowledge networks and demonstrate their thematic progress over time, which we call dynamic co-citation. For dynamic co-citation mapping, we divide the sample of 1626 articles into three sub-samples based on their publication year grouping in an expanding horizon. For instance, in this study, the first group includes articles published during 2000–2005, the second group during 2000–2010 and the final group during 2000–2019. Hereafter, these sub-sampled articles were analysed using co-citation. Typically, co-citation analyses can be of three types depending on the unit of analysis (1) journal co-citation, (2) author co-citation, and (3) document co-citation. In this study, we conduct document co-citation network analyses. This approach helps to identify the growth and knowledge development of the OVs research over time.

**Fig. 3:**
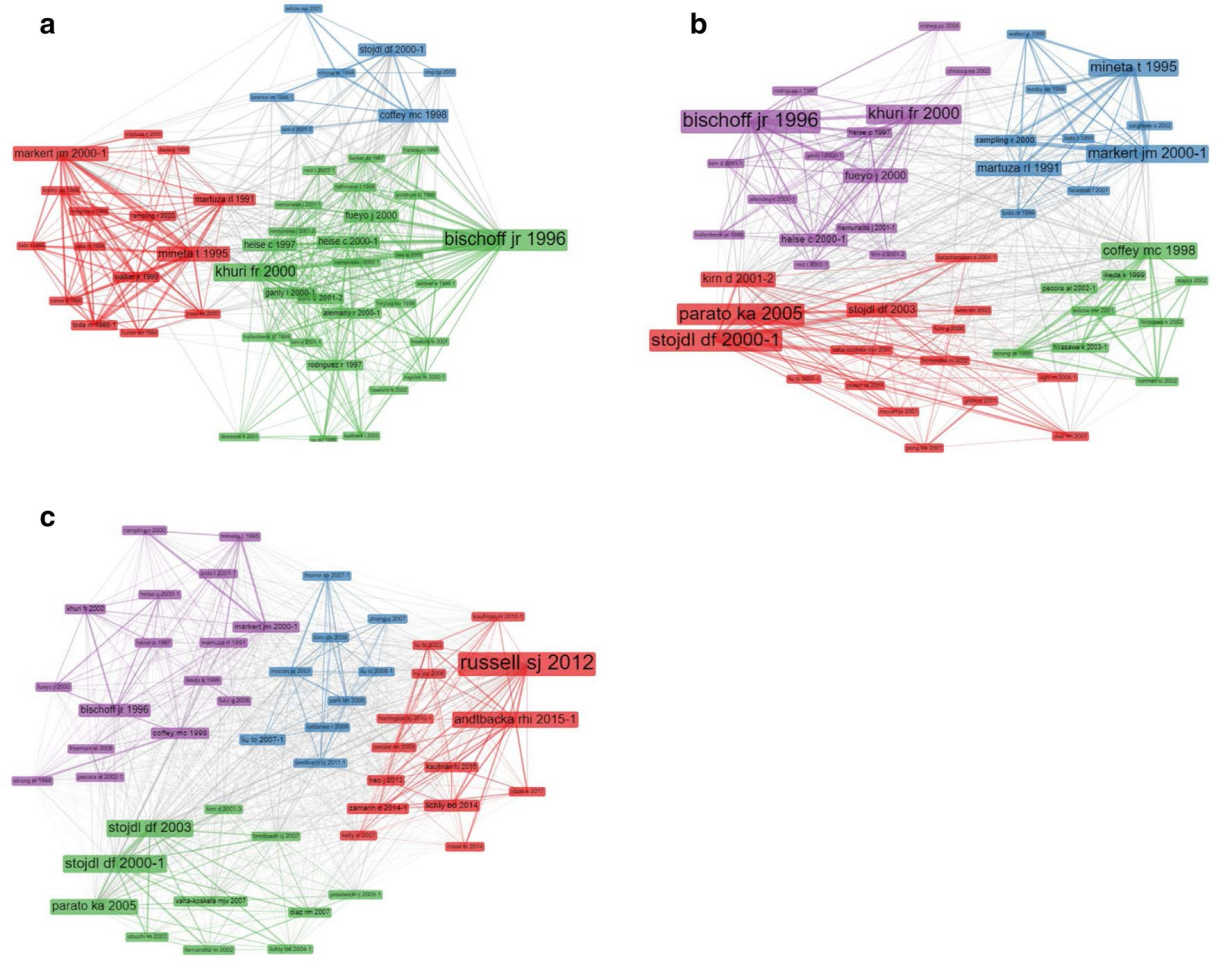
Dynamic co-citation mapping (2000–2019). (**a**) Co-citation 2000–2005, (**b**) Co-citation 2000–2010, (**c**) Co-citation 2000–2019

Figure 3 presents the change in the intellectual structure of the research field over time. In Fig. 3a, b and c, the number of documents analysed were 130, 469, and 1626, respectively. On the figures, each node represent an article. The size of the node presents the number of citations that the articles received. The line’s thickness represents the strength of co-citations ties. The link and proximity between two items identify the co-citation relationship. The colour of the node indicates the associated cluster of an article. Each node was specified by the first author name and publication year of the article. Association strength normalisation algorithm has been used in the Bibliometrix package to identify the clusters. Documents that are more often cited together are more likely to have a similar research topic, documents within the same cluster have a solid co-citation relationship and tend to portion similar research focus or theoretical basis.

### OVs intellectual structure during 2000–2005

As shown in Fig. 3a, the green cluster is one of the strongest among the three with 28 papers. In this cluster, Bischoff et al. [38], and Khuri et al. [39] had a stronger co-citation link. These articles report that several viruses have been engineered as oncolytic viruses. Bischoff et al. [38] reported adenovirus as one of the oncolytic viruses that is useful in treating human cervical carcinoma. They also reported the dl1520 (Onyx-015) is the first genome modified Conditionally Replicative Adenoviruses based on human adenovirus type 2/5 chimaera. Meanwhile, Khuri et al. [39] confirmed that the OXYN-15, the E1B-55 kDa gene-deleted adenovirus, showed an anti-cancer effect in head and neck cancer.

On the other hand, the red cluster is significant in terms of total co-citation link strength. This cluster is organised by 15 documents. Markert et al. [40] have the greatest connection strength and a strong link with both Mineta et al. [41] and Martuza et al. [42]. A diversity of oncolytic viruses are being studied in clinical trials, including gene deletion mutants such as adenovirus, herpes simplex virus. HSV is one of the most important oncolytic viruses and is extensively studied as anti-tumour agents, both experimentally and clinically. In this context, Mineta et al. [41] reported that HSV-1 G207 is effective in treating brain tumour in BALB/c mice. For this purpose, G207 has deletions at γ 34.5 (RL1) loci and an insertion of the *Escherichia coli* lacZ gene. The lacZ gene insertion inactivates the ICP6 gene (UL39) that encodes the large subunit of ribonucleotide reductase. Also, the first phase clinical trials by Market et al. [40] reported that HSV-1 G207, which belongs to the second generation genetically engineered HSV-1 mutants, has been shown to be effective in the brain tumour therapy.

Finally, the blue cluster is the smallest among the three containing seven articles. Stojdl et al. [21] has reported for the first time that vesicular stomatitis virus (VSV), a replication-competent oncolytic virus, is sensitive to the interferon response and is tumour-specific, owing to the deficiency of antiviral interferon signalling pathways in tumour cells. It is recognized to preferentially infect and lyse a wide-range of cancerous cells in pre-clinical models and in patients. Also, Coffey et al. [43] reported that to infect a cell with human Reovirus (respiratory enteric orphan) the virus needs to have an activated Ras signalling pathway. Furthermore, they demonstrated that this virus may have applicability in the treatment of cancer such as glioblastoma.

### Extended OVs intellectual structure during 2000–2010

In Fig. 3b, we examine the co-citation network of the second sub-sample studies published between 2000 and 2010. In comparison to Fig. 3a, the current figure has changes in cluster structures, although some remain stable. The blue cluster from Fig. 3a breaks down to two clusters in Fig. 3b—the red cluster with Stojdl et al. [21] at the centre and the green cluster with Coffey et al. [43] at the centre. The figure shows that the intellectual structure of the oncolytic viruses has extended and changed over time.

The green cluster contains nine documents about Reovirus, Newcastle disease virus, and Herpes simplex virus. All of these documents confirmed that Reovirus has potent activity against cancers such as colon cancer, breast cancer, ovarian cancer, and malignant gliomas in vitro, in vivo, and ex vivo. Hirasawa et al. [44] revealed that the use of systemic delivery of Reovirus agent concerning immune-suppressive drugs effectively prolongs animal survival. Pecora et al. [45] reported that in the first phase trial of PV701, a replication-competent strain of Newcastle disease virus can provide a novel and potentially important therapy for patients with solid tumours after intravenous administration.

The blue cluster (identical to the red cluster in Fig. 3a) has ten documents and almost all of the articles in this cluster focus on the oncolytic herpes simplex virus. Markert et al. [40], Mineta et al. [41], Martuza et al. [42], and Rampling et al. [46] have more document co-citations when compared to other studies. These studies confirmed the use of herpes simplex virus (ICP 34.5 null mutant 1716) in patients.

The purple cluster (identical to the green cluster in Fig. 3a) contains 15 documents, and it represents influential papers on adenovirus during 2000–2010. In this cluster, Khuri et al. [39], and Bischoff et al. [33] have more co-citations compared to the other authors. Meanwhile, we observed that several researchers worked on adenovirus in different clinical trial phases such as Reid et al. [47] and Khuri et al. [39] on Phase I, and Nemunaitis et al. [48] Phase II. Moreover, in the current cluster, we found that four review articles, Kirn [49], Kirn et al. [50], Alemany et al. [51], and Chiocca [52] have noticeable documents co-citations. Kirn [49] surveyed all the clinical trials about dl1520 (Onyx-015), which is the first genetically engineered agent to test on humans with an E1B-55 gene deletion. On the other hand, Alemany et al. [51] revealed types of conditionally replicative adenoviruses (CRAds) used as oncolytic agents till now.

The red cluster represents influential studies on OVs during 2000–2010, and consists of 16 documents. The majority of documents focus on applying vesicular stomatitis virus in the case of in vitro and in vivo studies on tumour cell lines model. We have also found that other OVs such as HSV, Measles, Adenovirus, and Vaccinia are potent anti-cancer agents. From these articles, four review articles by Kirn et al. [50], Parato et al. [53], Aghi and Martuza [54], and Liu et al. [55] explained the type of clinical trials of OVs. Vähä-Koskela et al. [56] investigated some of the recent additions to the panel of OVs including yaba-like disease virus, avian adenovirus, myxoma virus, bovine herpesvirus 4 (BHV-4), foamy virus, echovirus type 1, saimiri virus, sendai virus, feline panleukopenia virus, and the non-human coronaviruses. Also, Wein et al. [57] reported using preclinical and clinical data to validate the mathematical model of replication-competent adenovirus for cancer treatment.

### New developments during 2000–2019

As shown in Fig. 3a, documents in all three clusters (Red, Blue, and Green) are about different oncolytic viruses in the tumour cells model. Since the development in genetic engineering at the start of the 1990s, use of engineered oncolytic viruses for cancer therapy have increased [42]. Figure 3b represents the change in the intellectual structure of the oncolytic viruses during this period. Finally, Fig. 3c focuses on the new developments in oncolytic viruses during 2000–2019.

As shown in Fig. 3c, the purple cluster is one of the strongest among others, with 16 documents. In this cluster, the majority of the documents repeat from Fig. 3a and b, except Toda et al. [58] and Freeman et al. [27]. Freeman et al. [27] revealed that the NDV-HUJ strain of Newcastle disease virus had shown good tolerability in phase I/II clinical trial for the treatment of glioblastoma multiforme (GBM), as well as other cancers. Also, the authors confirmed that lentogenic NDV strains should also be surveyed in patients with lower-grade gliomas. Todo et al. [58] reported the use of soluble B7-1 in the context of oncolytic HSV for immune gene therapy and is clinically suitable in in-situ cancer vaccination.

The red cluster has 14 documents and concentrates on HSV, NDV, and vaccinia virus. Six of the papers in this cluster explored the HSV. In this cluster, six review articles—Kelly and Russell [59], Harrington et al. [60], Russell et al. [61], Lichty et al. [62], Miest and Cattaneo [63], and Kaufman et al. [64] had the most document co-citation. Russell et al. [61] and Andtbacka et al. [65] were the most co-cited studies of this cluster. Russell et al. [61] reported that oncolytic virotherapy is a novel therapeutic modality that uses replication-competent viruses against cancers. Additionally, Andtbacka et al. [65] revealed that T-VEC is the first oncolytic immunotherapy to display therapeutic profit against melanoma in phase III clinical trial. Also, it can represent a potential advance treatment for patients with injectable metastatic melanoma. However, in the current cluster, all of the documents are new document in co-citation, and these documents do not include in Fig. 3a or b. During this period (2000–2019) some development includes—the possibility of a single-shot virotherapy treatment, recognition of new drugs to speed up intratumoral virus diffusion, augmentation of the immunotherapeutic action of OVs, and clinical confirmation of a critical threshold of virus in the blood for vascular delivery and virus replication within the tumour [61].

The green cluster contains 11 documents among which some repeats from Fig. 3b except Obuchi et al. [66], Lichty et al. [67], Breitbach et al. [23], and Prestwich et al. [30]. Out of 11 documents, four are review studies. These studies surveyed three phases of the clinical trial of oncolytic viruses in different tumour cell models. Lichty et al. [67] described VSV as a therapeutic oncolytic virus. On the other hand, Prestwich et al. [30] discussed Reovirus as it generates adaptive antitumor immunity in vitro and in vivo studies. Additionally, Breitbach et al. [23] revealed that unappreciated and unanticipated interaction between VSV and vaccinia virus, and inflammatory response in the tumour.

The last cluster is the blue one with nine articles out of which five are review studies. This cluster consists of new studies except McCart et al. [68]. Liu and Kirn [69], Liu and Kirn [70], and Cttaneoet al. [71] reviewed some phases of the clinical trial of oncolytic viruses in different tumour cell models. Thorne [72] discussed oncolytic viruses, eukaryotic cells, and attenuated bacteria and the mechanisms to deliver them systemically to tumours, including in case of micro metastases. In this cluster, other studies by McCart et al. [68], Zhang et al. [73], and Park et al. [74] were focused on the oncolytic Poxviridae family. Moreover, for the first time, Breitbach et al. [75] reported that VSV is able to infect tumour neovasculature in vivo, but normal tissue vascular endothelium is resistant to the virus infection.

## Discussion

This study discusses a diversity of OVs that has potential for types of anti-cancer therapy. Since its development, genetically engineered OVs are studied as a suitable alternative to non-engineered viruses (wild-type). As cancer therapy field has changed, OVs have an improved therapeutic index. Although the accumulated data has impressive recent development in cancer therapy using oncolytic viruses, it is prevented at many levels and may require assistance to reach full efficacy. Several studies also reported that oncolytic virotherapy could be utilised for antitumor treatment through different combination strategies such as chemotherapy, radiotherapy, systemic immunotherapies, etc. [7, 56, 76, 77]. This combination has resulted in enhance apoptosis induction and showed significant result in a wide range of tumour models. Most popular drugs that fall into the classes are cyclophosphamide (CPA) doxorubicin, camptothecin (CPT), 5-fluorouracil (5-FU), ganciclovir (GCV), cisplatin, mitomycin C (MMC), paclitaxel, carboplatin, rapamycin, rituximab, and docetaxel [78–80]. Additionally, Vähä-Koskela et al. [56] reported OVs such as adenovirus in combination with chemotherapy, have been confirmed as a standard therapy to treat refractory nasopharyngeal cancer in China.

During the last decade, the fast extension of global OVs research resulted in benefits to the population. Son et al. [81] revealed the combination of OVs with various therapies leads to improved infection efficiency and increased antitumor effects. They suggested that the cytopathic effects of measles and mumps viruses’ combination (MM) can improve the antitumor activity and tumour cell killing in vitro and in vivo. For further research, the study proposed explaining the ability of the antitumor reactions of oncolytic viruses and combination. Moreover, the recent study by Al‑Shammari et al. [82] confirmed that combination therapy of oncolytic Newcastle disease virus and *ribes rhizomes* extract enhanced the anticancer activity. Interestingly, the study proposes the combination of OVs with herbal therapy which may result in novel anticancer therapy. Several studies measured the treating advantage of radionuclide therapy in combination with oncolytic viruses and external beam radiotherapy. The results showed that OVs combined with expressing the sodium iodide symporter (NIS) in various tumour models could restrict tumour growth and increase survival [83–86].

As shown in Table 4, North America, including the USA and Canada, has a strong effect on the oncolytic virus research, while European institutions, including the UK, Germany, and Finland, also play a prominent role. Demir et al. [87] revealed that countries with significant economic potency such as United States, Japan, the United Kingdom, Canada, Australia, and China could be the most impactful in terms of the number of publications which is in line with our results.

The USA, being the leading high-tech power upon the offset of the Second World War, leads many global research arenas. Such an issue was also considered in our study in terms of the numbers of articles, institutions, and scientists, showing that USA scientists have contributed greatest influence on virotherapy advancement. The science and technology operational impact policy, the rich financial support was driven from public foundations and private enterprises, and the implementation of new or better devices developed in the USA serves as the potential bases for the USA's most considerable contribution [88].

## Limitation

The first publication regarding OVs’ role in clinical or preclinical studies, was published in 1912 [59]. The database used in this study, WoS, only list publications from 1980 which left behind previous studies. But as a novel therapy, most development in OVs has occurred recently; therefore, recent studies would give a better overview of the current state of the study domain. This study did not cover scientific literature from Scopus and PubMed, and only journals with an impact factor (IF) are indexed in the WoS database. While this exclude studies published in the journals without an IF, eventually results a review of only high-quality studies. Moreover, studies confirmed the use of only the WoS for the bibliometric study exerts more reliable results than other databases of peer-reviewed scientific literature such as Scopus and PubMed [87].

## Conclusion

This study provides a holistic review of the present body of literature focusing on oncolytic viruses as one of the potential therapies in cancer treatment. This review maps the intellectual structure development of OV research during 2000 to 2020 using dynamic co-citation analysis. From the content analysis of the most co-cited studies, adenovirus, herpes simplex virus, Reovirus, and Newcastle disease virus have shown potent activity on the treatment of several cancer types. Based on citation analysis, the developed countries were the most productive in publications on OVs. Conducting multinational research studies would help other countries to enter into the research domain and get the possible benefit. The findings of this bibliometric review provide beneficial knowledge for clinicians, especially for oncologists and researchers, for exploring OVs considering development trends.

## Data Availability

We have attached the bibliography data analyzed in this study in zip format for consideration.

## Abbreviations

OVs: Oncolytic viruses
GM: Genetically modified
NDV: Newcastle disease virus
SVV: Seneca valley virus
VSV: Vesicular stomatitis virus
HSV: Herpes simplex virus
WoS: Web of Science
T-Vec: Talimogene laherparepvec
Pexa-Vec: Pexastimogene devacirepvec
